# Nutritional profile of newborns with microcephaly and factors associated with worse outcomes

**DOI:** 10.6061/clinics/2019/e798

**Published:** 2019-10-14

**Authors:** Samira Fernandes Morais dos Santos, Fernanda Valente Mendes Soares, Andrea Dunshee de Abranches, Ana Carolina Carioca da Costa, Saint Clair dos Santos Gomes-Júnior, Vania de Matos Fonseca, Maria Elisabeth Lopes Moreira

**Affiliations:** Instituto Nacional de Saude da Mulher, da Crianca e do Adolescente Fernandes Figueira, Fundacao Oswaldo Cruz (IFF/Fiocruz), Rio de Janeiro, RJ, BR

**Keywords:** Zika Virus, Microcephaly, Nutritional Status, Newborn

## Abstract

**OBJECTIVE:**

To describe the nutritional profile of newborns with microcephaly and factors associated with worse outcomes during the first 14 days of life.

**METHODS:**

This investigation is a longitudinal, descriptive study carried out in 21 full-term neonates exposed vertically to the Zika virus and hospitalized in a neonatal intensive care unit from February to September 2016. Patients receiving parenteral nutrition were excluded. Data analysis was performed using a generalized estimating equation model and Student’s t-test to evaluate the association between worsening weight-for-age z-scores and independent clinical, sociodemographic and nutritional variables during hospitalization, with *p*<0.05 indicating significance.

**RESULTS:**

During hospitalization, there was a decrease in the mean values of the weight-for-age z-scores. The factors associated with worse nutritional outcomes were symptomatic exposure to the Zika virus, low maternal schooling, absence of maternal income and consumption of infant formula (*p*<0.05). Calcification and severe microcephaly were also associated with poor nutritional outcomes. Energy and macronutrient consumption remained below the recommendations and had an upward trend during hospitalization.

**CONCLUSION:**

The presence of cerebral calcification, the severity of microcephaly and symptomatic maternal exposure to Zika virus affected the nutritional status of newborns. In terms of nutritional factors, human milk intake had a positive impact, reducing weight loss in the first days of life. Other known factors, such as income and maternal schooling, were still associated with a poor nutritional status.

## INTRODUCTION

In November 2015, Brazil declared a national public health emergency due to a continued increase in cases of microcephaly. The Ministerio da Saude (Brazil) confirmed the association between these cases and Zika virus (ZIKV) infection during pregnancy, since the virus was detected in samples of brain tissue from these newborns (1-3). This association and its consequences drew worldwide attention, drawing international concern for microcephalic newborns and their deficits in motor development, cognition, and auditory and visual abilities, among other associated changes, as well as the social impact on their families (4,6).

In this setting of many uncertainties, the various aspects of microcephaly must be clarified, including questions related to nutrition. Nutritional assessment appears to be another perspective of child health care that can be added to their primary care (7), mainly because there are still few studies related to nutrition for these babies.

As other studies have already established (8-10), children with neurological impairment, such as infants with microcephaly, are a nutritional risk group due to several factors that contribute to the frequent presence of malnutrition. According to the Ministry of Health (3), the main nutritionally relevant findings related to Congenital ZIKV syndrome are hyperirritability, hyperexcitability, difficulty suckling and swallowing. These factors may compromise nutrition, growth and development depending on the type and severity of the clinical picture. Thus, these newborns require further investigation and intervention because the first thousand days of life are the period of the greatest brain plasticity, and nutrition can impact neurocognitive development and growth and reduce the lifelong risk of various chronic diseases and comorbidities (11).

To characterize this population in order to inform measures that minimize the effects of inadequate nutrition, this study aims to describe the nutritional profile of newborns with microcephaly and the factors associated with worse outcomes in the first 14 days of life.

## METHODS

In order to understand the nutritional status of ZIKV-exposed children and investigate related factors, a longitudinal descriptive study was carried out under the project “Vertical exposure to Zika virus and its consequences in child neurodevelopment”, conducted at the Instituto Nacional de Saude da Mulher, da Crianca e do Adolescente Fernandes Figueira - Fundacao Oswaldo Cruz (IFF/FIOCRUZ), Rio de Janeiro, RJ, BR. The aforementioned project was approved by the IFF/FIOCRUZ Research Ethics Committee (CAAE 52675616.0.0000.5269) and is ongoing, continuing to recruit and follow infants and children in the cohort. Assistance to this population is provided periodically by a multiprofessional team (doctors, nurses, psychologists, social workers, physiotherapists, nutritionists). Some papers from this group have already been published (12-18).

We included all newborns with gestational age between 37 and 42 weeks at birth (assessed from the date of the last menstrual period or ultrasonography in the first trimester of pregnancy) admitted to the IFF/FIOCRUZ Department of Neonatology Unit from February to September 2016 with a diagnosis of microcephaly due to exposure to ZIKV. Microcephaly was diagnosed according to a measured head circumference at birth (HC) less than two (-2) standard deviations below the mean (according to the Growth Curves of the World Health Organization - WHO, 2006 (19)) and/or medical findings on imaging studies of the central nervous system (transfontanelle ultrasonography and tomography). Exposure to the Zika virus was defined as having a mother who reported symptoms of ZIKV (skin rash, fever associated with arthralgia, myalgia, nonpurulent conjunctivitis or headache) during pregnancy and/or a positive polymerase chain reaction test (RT-PCR) result during the gestational period. Newborns who received parenteral nutrition therapy during hospitalization were excluded.

Information was obtained from cohort data and patient records and recorded on the research form. We collected anthropometric data (daily weight, HC and height measurements), altered brain imaging (the presence of calcification, ventriculomegaly, cerebellar hypoplasia, hydrocephalus and mega cisterna magna) and maternal history of symptomatic exposure to the Zika virus.

Data obtained from the Live Birth Certificate were maternal age (years), level of schooling (years of study), number of prenatal consultations and usual occupation. The usual maternal occupation was divided into two income categories: the no income category comprised mothers who worked within the home and/or were students, and the with income group comprised mothers whose usual occupation took place outside the home and/or generated income.

Regarding newborn feeding, data on the type of milk consumed (infant formula / human milk), the volume ingested (milliliters - mL) and the route of administration (oral, nasogastric, orogastric) were collected daily. This information was used to calculate the total energy value (calories per kg, proteins (g/kg), carbohydrates (g/kg) and lipids (g/kg)) administered effectively to these newborns.

To calculate the nutritional intake from infant formula, we considered the nutritional information on the label of each product. For human milk (HM) pasteurized or breastfeeding, we used the values published by Vieira et al. (20) and the Ministry of Health (21), respectively.

The institution in which the study was carried out (IFF/Fiocruz) is accredited by the Baby-friendly Hospital Initiative and has a Human Milk Bank unit (BLH). In this way, the institution prioritizes breastfeeding; if breastfeeding is not possible, infants receive expressed milk from their mothers or from the BLH. If HM is not indicated for clinical reasons or is not available, infants are given an age-specific infant formula.

To estimate energy and macronutrient needs, we used the Estimated Energy Requirement for the 0-3 month age group and the Adequate Intake for carbohydrates, lipids and proteins for the 0-6 month age group, proposed by the Dietary Reference Intake (DRI) published by the Institute of Medicine (22). We determined the appropriate nutritional therapy using the recommended percentages of energy and macronutrients and administered it daily to the newborns.

The patients were classified using the following indicators: weight for age (W/A), height for age (H/A), body mass index (BMI) for age (BMI/A), weight for height (W/H) and HC for age (HC/A). These indices were stratified as follows: z-scores <-2 (low W/A, BMI/A, H/A and W/H), z-scores ≥-2 and ≤2 (adequate W/A, BMI/A, H/A and W/H), z-scores >2 (high W/A) and HC/A >2, and z-scores <-3 (severe microcephaly) (23), according to the 2006 WHO Growth Curves (19).

The data were descriptively analyzed using measurements of frequency, central tendency (mean and median) and dispersion (standard deviation). We used a generalized estimating equation model to compare W/A z-scores during hospitalization according to the following variables: maternal exposure to the Zika virus; maternal schooling (years of study); maternal income (usual paid or unpaid occupation); route of diet administration (naso/orogastric or oral); type of milk consumed (HM or infant formula); presence or absence of calcification(s); ventriculomegaly; cerebellar hypoplasia; mega cisterna magna; hydrocephalus and severe microcephaly. On days when greater variations of the mean z-score values were detected, we applied Student’s t-test to verify the associations between the variables and the outcome for these specific days. All analyses considered *p*≤0.05 significant.

The data were recorded and stored on an Excel^®^ spreadsheet and analyzed in SPSS^®^ 21. Z-scores were calculated using Antho^®^ software (24).

## RESULTS

Regarding maternal characteristics, 90.5% of the mothers were aged between 18 and 34 years, 61.9% had symptoms of ZIKV during pregnancy, and 80% had no partner. The mean hospitalization time of the 21 infants included in the study was 11.7 days (data not shown in Table 1). Of these, 81% had severe microcephaly, and 85.7% presented adequate weight for age z-scores at birth. Regarding brain imaging alterations, the most common ones were ventriculomegaly and calcification (Table 1).

**Figure 1<AQ>Please:**
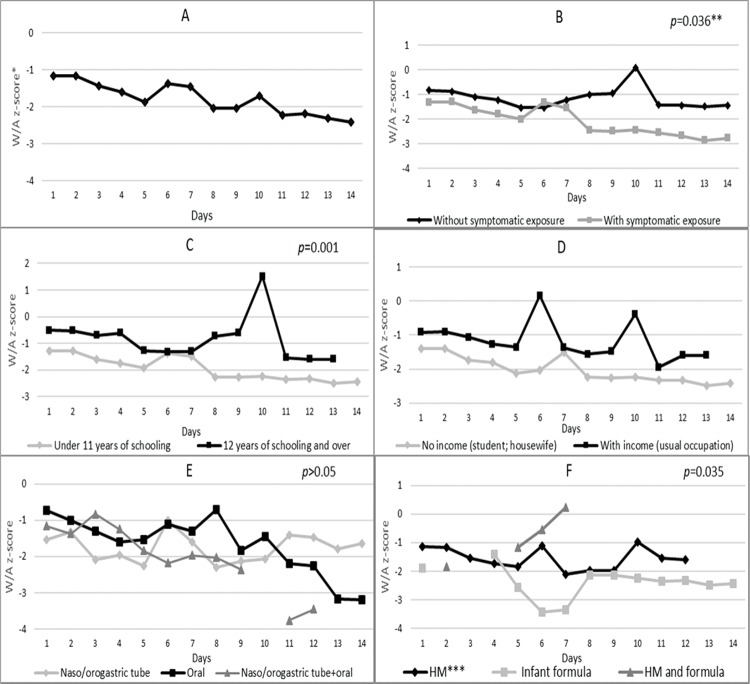
Nutritional status (weight-for-age z-score) of microcephalic newborns at IFF/Fiocruz, Rio de Janeiro, Brazil, 2016: variation according to A - days of hospitalization; B - categories of exposure to Zika virus; C - maternal level of schooling; D - maternal income; E - diet administration route; and F - type of milk consumed during hospitalization. *W/A: weight-for-age; **generalized estimating equation model; ***HM: human milk; ****Broken lines in some charts are due to the lack of a newborn in that category on that day of hospitalization.

Figure 1 (Chart A) reveals that the patients had a mean weight-for-age z-score of -1.17 on the first day of hospitalization, which decreased to -2.03 on day 8 and reached -2.42 on day 14. Nutritional status according to maternal exposure to the Zika virus (Chart B) indicates that the newborns of mothers without symptoms of the virus had higher mean z-scores than those whose mothers had exposure symptoms. This difference was statistically significant (*p*=0.036) over the evaluated period.

Newborns of mothers with 12 years of schooling and over had higher mean z-scores than those born to mothers with 8-11 years of schooling (Figure 1, Chart C). This value inequality was statistically significant (*p*=0.001) on all evaluated days and, in isolation, on the 10^th^ day (*p*<0.000), when the first group and the second group showed z-scores of 1.52 and -2.25, respectively.

Neonates who were fed orally showed progressively declining scores from the 10^th^ day and a mean z-score of -3.20 on the 14^th^ day, which was lower than the z-scores for infants fed by the nasogastric or orogastric route (-1.65) (Figure 1, Chart E). From the 5^th^ day onwards, the mean z-scores of newborns who consumed only infant formula tended to be smaller than those of infants who consumed only HM- Figure 1, Chart F. This difference was statistically significant (*p*=0.035) on all evaluated days. Starting on approximately the 4^th^ day, the group that consumed only infant formula had a longer hospitalization period than the group that consumed only HM during this period.

**Figure 2:**
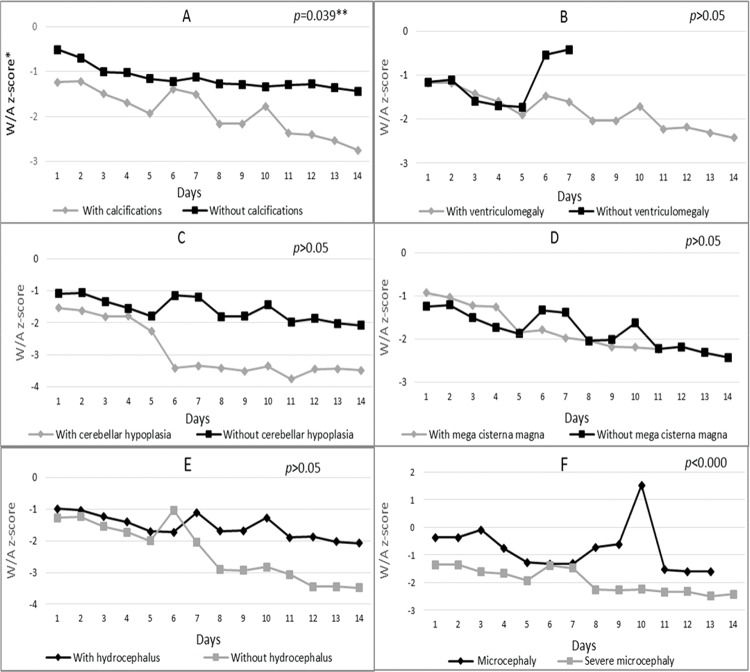
Nutritional status variation (weight-for-age z-score) according to the presence or absence of A-Calcifications, B-Ventriculomegaly, C-Cerebellar hypoplasia, D-Mega cisterna magna and E-Hydrocephalus and F-Degree of microcephaly during the hospitalization of microcephalic newborns at IFF/FIOCRUZ, Rio de Janeiro, Brazil, 2016. *W/A: weight-for-age; **generalized estimating equation model; ***Broken lines in some charts are due to the lack of newborns in the category on the day of hospitalization.

Regarding variation in z-scores according to altered brain imaging (Figure 2), Chart A shows that infants with calcification had significantly lower mean z-scores (*p*=0.039) on all days compared to infants without this change. On the first day of hospitalization, the first group had a mean z-score of -1.24, and on day 14, this value dropped to -2.75. The group without calcification had mean z-scores of -0.51 on day 1 and -1.44 on day 14. The same results were found for newborns with ventriculomegaly and cerebellar hypoplasia, who showed predominantly smaller mean z-scores when compared to newborns without these alterations (Figure 2, Charts B and C).

Children with mega cisterna magna (Figure 2, Chart D) had lower mean z-scores (W/A) from the 5^th^ day of hospitalization (-1.87) compared to newborns without a neurological condition (-1.84 on the 5^th^ day) (Figure 2, Chart D). Regarding the presence of hydrocephalus (Figure 2, Chart E), newborns with this alteration had higher mean z-scores on the first day of hospitalization (-0.98) and on the 14^th^ day (-2.07) compared to newborns without hydrocephalus (-1.26 and -3.48 on the first and 14^th^ days, respectively).

During hospitalization, newborns born with severe microcephaly had lower mean z-scores compared to the less-severe group (Figure 2, Chart F). This difference was statistically significant (*p*<0.000) throughout hospitalization.

**Figure 3:**
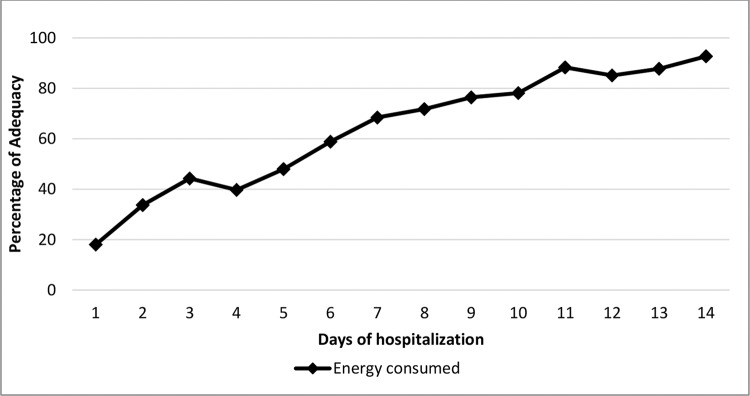
Percentage of adequate consumption of the estimated energy requirements by microcephalic newborns during hospitalization at IFF/FIOCRUZ, Rio de Janeiro, Brazil, 2016. Percentage with adequate consumption; Days of hospitalization; Energy consumed.

Regarding whether nutritional therapy met the infants’ estimated needs, energy intake maintained an upward trend throughout the evaluated period (except on day 3) - Figure 3. However, it only met the recommended values on the 14^th^ day. The consumption of carbohydrates, proteins and lipids showed the same trend and was below the recommendations from the first to the 14^th^ day.

## DISCUSSION

Symptomatic maternal exposure to ZIKV negatively influenced the nutritional status of newborns in the first 14 days of life, with changes observed in weight for age (z-score (W/A)). Another study (18) involving the same cohort of infants exposed to ZIKV during pregnancy without microcephaly showed that these infants were smaller, lighter and had a lower percentage of body fat in the first two months of life compared to nonexposed infants. This finding shows that maternal ZIKV exerts a strong impact on the worsening nutritional status of babies born with and without microcephaly.

Calcification and severe microcephaly were associated with lower z-scores during hospitalization. Patients who had other alterations, such as ventriculomegaly, mega cisterna magna and cerebellar hypoplasia, as identified in studies that addressed ZIKV Congenital Syndrome (25,26), also had lower mean z-scores, although the difference was not statistically significant. However, newborns with hydrocephalus, a condition characterized by the accumulation of cerebrospinal fluid in the cerebral ventricles and the subarachnoid space (27), showed predominantly higher mean z-scores during the period evaluated. The accumulated cerebral fluid increases body weight; consequently, a higher W/A z-score does not necessarily reflect a better nutritional status. In addition to increasing the severity of symptoms among newborns, microcephaly associated with other altered brain imaging was associated with worse nutritional status. These findings indicate the need for more specific and individualized care for this population because small changes in the W/A z-scores can affect the growth and development of these infants (28).

Del Campo et al. (25) studied 83 infants with Congenital ZIKV Syndrome with and without microcephaly and found that 81.3% had an adequate W/A (z-score ≥2 and ≤-2) at birth. In our study, this parameter showed a similar prevalence. These data indicate that most of these babies are born with no weight impairment and that their worsened nutritional status may be due to clinical and environmental issues.

Neonates born to mothers with up to 11 years of schooling and no occupational income had significantly lower z-scores than those whose mothers had higher schooling (over 12 years of schooling) and an occupational income, likely due to maternal nutritional conditions during the gestational period. Other authors (29) found similar results; they found an association between low birth weight infants with no comorbidities and lower maternal schooling and economic levels. These factors were related to lower levels of prenatal care.

Another important social factor that may be related to the changes in the nutritional status of newborns was that 80.9% of mothers were single. A study (30) of 40 women affected by the Zika virus epidemic showed cases of abandonment by their partners (including when they were informed of the diagnosis of microcephaly in the fetus), which contributed to worse life conditions for these women in terms of poverty. Environmental conditions have direct repercussions on the health of newborns in the short and long term (11). Children are more likely to maximize their potential for growth and development when they are well nourished, cared for and have learning opportunities from birth (31).

Nutrition plays a fundamental role in the favorable evolution of the patient’s clinical condition and consequently in his or her hospital stay (32). In terms of our findings, it is worth mentioning that the type of milk the infants consumed was associated with nutritional status. From the 5^th^ day onwards, the mean z-scores of neonates who consumed only HM (via maternal breastfeeding and/or expressed maternal milk and pasteurized milk from the milk bank) tended to be higher than those of infants who only consumed infant formula. The findings of Davanzo et al. (33) also showed that HM had a positive impact, reducing neonatal weight loss compared to infant formula only; their study, however, included breastfed neonates without microcephaly. The ability to maintain breastfeeding in infants with microcephaly should be investigated because these infants generally present dysphagia and difficulty suckling during the first months of life, along with an elevated risk of reflux and gagging (3,5,34). These characteristics hinder breastfeeding and its maintenance and increase the need for frequent breastfeeding evaluations and interventions.

Still, in this context, patients with more severe clinical conditions in our study may have consumed only infant formula, not having access to HM and its countless benefits (35). These data encourage reflection on nutritional practices and milk selection and show the importance of daily nutritional and clinical evaluations of nutritional therapy.

During hospitalization, energy and macronutrient intake showed an upward trend but did not met the estimated requirements during the period evaluated. Given that nutritional deficiencies can burden the health of already compromised children (36), early interventions aimed at ensuring adequate nutritional intake may benefit these infants in the short and long term. Nutritional care provides an opportunity to maximize the outcomes of newborns as it is a relatively controllable factor in neonatology. Certain nutrients, when administered early, have a high impact on brain development, corroborating the hypothesis that nutritional support can focus on disease prevention by providing essential nutrients, consistently evaluating nutritional status, and providing early intervention for deficiencies when they exist.

Although all microcephalic newborns exposed to ZIKV were considered during the study period, this sample was small (21) and therefore not ideal for hypothesis testing. Thus, the goal of a descriptive, relevant and timely study in this initial stage of research is to produce hypotheses for future comparisons with other populations that may serve as controls. Babies with microcephaly associated with ZIKV most commonly present hyperirritability, hyperexcitability, and epileptic seizures. These factors and physical restrictions associated with their clinical condition must be further investigated to determine the actual metabolic expenditure of these children, which may differ from that of same-age infants without neurological impairment. Studies that address these factors would help mothers and health professionals to prevent future obesity in these children.

In conclusion, the present study indicates that the presence of cerebral calcification, severe microcephaly and symptomatic maternal exposure to ZIKV affect the nutritional status of newborns. From a nutritional aspect, HM intake had a positive impact on reducing weight loss in the first days of life. Factors such as maternal income and schooling are still associated with worse nutritional status. These findings highlight the importance of deepening the knowledge of clinical and nutritional factors that may negatively affect the current and future health of infants with microcephaly and support the need for individualized nutrition and the incentivization of breastfeeding.

## AUTHOR CONTRIBUTIONS

Santos SFM was responsible for the manuscript writing, data collection and interpretation of results. Soares FVM was responsible for the study co-orientation. Abranches AD was responsible for the manuscript writing and interpretation of results. Costa ACC and Gomes-Júnior SCS were responsible for the statistical analysis of data. Fonseca VM and Moreira MEL were the advisors who interpreted the results.

## Figures and Tables

**Table 1:** Demographic, socioeconomic and clinical characteristics of mothers and their microcephalic newborns hospitalized at the IFF/Fiocruz, Rio de Janeiro, from February to September 2016.

Variable	Category	n	%
**Maternal demographic, clinical and socioeconomic characteristics**			
Age (years)	<18	2	9.5
	18-34	19	90.5
Schooling (years of study)	8-11	18	85.7
	≥12	3	14.3
Maternal income	With income (usual occupation)	11	52.4
	No income (student/housewife)	10	47.6
Symptomatic exposure to Zika virus during pregnancy	Yes	13	61.9
	No	8	38.1
Marital status	With partner	4	20
	Without partner	16	80
Prenatal care consultations	<6	7	38.9
	>6	11	61.1
Marital status	With partner	4	20
	Without partner	16	80
Prenatal care visits	<6	7	38.9
	>6	11	61.1
**Birth-related**			
Gender	Male	9	42.9
	Female	12	57.1
Birth type	Normal	15	71.4
	Cesarean	6	28.6
Apgar 5^th^ minute	≤7	0	0
	>7	21	100
Weight-for-age z-score	<-2	3	14.3
	≥- 2 and ≤2	18	85.7
Height-for-age z-score	<-2	3	14.3
	≥- 2 and ≤2	17	80.9
	>2	1	4.8
BMC^a^-for-age z-score	<-2	5	23.8
	≥- 2 and ≤2	15	71.4
	>2	1	4.8
Weight-for-height z-score	<-2	5	23.8
	≥- 2 and ≤2	16	76.2
HC^b^-for-age z-score	<-3	17	81
	>-3	4	19
**Altered brain imaging**			
Hydrocephalus	Yes	7	33.3
	No	14	66.7
Calcification	Yes	19	90.5
	No	2	9.5
Ventriculomegaly	Yes	19	90.5
	No	2	9.5
Cerebellar hypoplasia Mega cisterna magna	Yes	3	14.3
	No	18	85.7
	Yes	5	23.8
	No	16	76.2

a BMI - body mass index; ^b^ HC - head circumference.
